# Intra-Muscular Injections of Mercury in the Treatment of Syphilis

**Published:** 1898-02-26

**Authors:** 


### INTRA-MUSCULAR INJECTIONS OF MERCURY IN THE TREATMENT OF SYPHILIS.

Surgeon-Major Lambkin again draws attention to the advantages to be derived from intra-muscular injection of mercury in the treatment of syphilis among soldiers. He points out that the soldier on first getting syphilis is treated in hospital, but he is only kept there while the more active primary and secondary signs of the disease continue. It is, he says, impossible to keep him in long enough to ensure a cure, while, on the other hand, it is impossible to carry on treatment by the more ordinary methods after he has returned to duty. The injections, however, only require to be used once a week, and take the soldier from his duty not more than half an hour each time. This seems to us to have an important bearing on civil practice. The treatment of syphilis among the civil population is notoriously unsatisfactory. On the one hand, it is neglected by the careless; on the other hand, it is far too often overdone by men who, having got hold of a prescription which they fancy suits them, go on taking it in season and out of season much to their own detriment. If only as a reason and excuse for a weekly inspection the injection method would be worth much; but if we are to accept the results obtained by Surgeon-Major Lambkin, as representing what may be expected in general practice, there is much to be said for it, both in regard to efficiency and freedom from inconvenience. A great point in its favour is that the practitioner is able to know exactly what is being done, for the simple reason that he does it himself.

It is now a good many years since this mode of treatment was introduced. Many preparations of mercury have been employed in carrying it out: Calomel, yellow oxide, peptonate, grey oil (an emulsion of mercury in lanoline and olive oil), perchloride, bicyanide, salicylate, and sozoiodol of mercury; and on the whole the general consensus of opinion among those who have practised injection is in favour of its efficiency; but certain objectionable effects have been met with, especially pain and inflammatory reaction, and even abscess.

Professor Lang is strongly in favour of grey oil; while Lewin, who has had a very large experience, speaks highly of the perchloride. It is to be noted, however, that in the administration of the perchloride the injections are very frequently repeated, sometimes as often as three times a day, while the insoluble preparations are given at much longer intervals.

Surgeon-Major Lambkin has lately been using the following solution: Sodii iodidi. gr. 10; hydrarg. sozoiodol, gr. 5; aquæ distill. ♏200. Of this ♏10 to ♏15 is used as an injection. It causes more pain at the time of injection and for some time afterwards than the "cream," and it does not seem to have quite so rapid an action. Still, as it possesses none of the disadvantages of the "cream," he has no hesitation in recommending it for general use. The "cream," however, is the preparation on which most of his experience seems to have been gained. It is very like the "grey oil" used by Professor Lang, and is composed of: Hydrarg. ʒi.; lanolin. pur., ʒii.; ol. carbol (2 per cent.), ʒi. Maximum dose ♏10.

In Lang's preparation the lanolin is first dissolved in chloroform, which is then allowed to evaporate; a certain quantity of chloroform, however, seems to remain in combination with the oily mass. This compound is then triturated with the mercury until all the chloroform has disappeared and the mercury and lanolin are thoroughly blended. This mass is then diluted with olive oil to the required degree.

The difficulties which Surgeon-Major Lambkin has met with in the use of the cream seem to have arisen principally from its consistency varying according to temperature, and from the tendency of the mercury to separate when the cream becomes too fluid. The temperature of one's consulting-room need not vary greatly, and by adopting Lang's plan of preparation and diluting as required, these difficulties ought not to be insuperable. If treatment is to be persevered with after symptoms cease, it is very important that it should be as painless as possible.

